# Clinical results of proton beam therapy for elderly patients with non-small cell lung cancer

**DOI:** 10.1186/s13014-018-0967-4

**Published:** 2018-02-05

**Authors:** Takashi Ono, Tatsuya Nakamura, Hisashi Yamaguchi, Yusuke Azami, Kanako Takayama, Motohisa Suzuki, Hitoshi Wada, Yasuhiro Kikuchi, Masao Murakami, Kenji Nemoto

**Affiliations:** 1 Department of Radiation Oncology, Southern Tohoku Proton Therapy Center, 7-172, Yatsuyamada, Koriyama, Fukushima Japan; 2 Department of Radiation Oncology, Yamagata University Faculty of Medicine, 2-2-2, Iida-Nishi, Yamagata, Japan

**Keywords:** Elderly, Non-small cell lung cancer, Protons, Pneumonitis

## Abstract

**Background:**

The purpose of the present study was to evaluate retrospectively the efficacy and safety of proton beam therapy for elderly patients (≥80 years of age) with non-small cell lung cancer.

**Methods:**

Patients diagnosed with T1–4 N0 M0 non-small cell lung cancer and treated with proton beam therapy between January 2009 and 2015 were recruited from our database retrospectively. Toxicity was evaluated using The Common Terminology Criteria for Adverse Events version 4.0.

**Results:**

Thirty-five patients, including 25 (71%) with clinically inoperable lung cancer, were administered proton beam therapy. The median age was 82 years (range: 80–87 years), and the median follow-up time was 34 months (range: 10–72 months). The median dose of proton beam therapy was 80.0 Gy relative biological effectiveness (RBE) (range: 60.0–80.0 Gy [RBE]), and all patients completed the treatments. All patients were followed for at least 23 months or until their death. The 3-year overall survival rate was 67.2% (90.0% in patients with operable lung cancer, and 58.2% in those with inoperable lung cancer). The 3-year local control rate was 86.5%. Two patients presented with grade 2 pneumonitis. The occurrence rate of grade 2 pneumonitis was significantly correlated with a high lung V20 (*p* = 0.030), and a high mean lung dose (*p* = 0.030), and a low ratio of lung volume spared from 0.05 Gy (RBE) dose (total lung volume minus lung volume irradiated at least 0.05 Gy [RBE]) (*p* = 0.030). However, there were no cases of grade 3 or higher radiation pneumonitis.

**Conclusions:**

This study suggests that the proton beam therapy was feasible for elderly patients with non-small cell lung cancer and can be considered as one of the treatment choices for elderly patients with lung cancer.

## Background

Lung cancer was the most frequently diagnosed cancer worldwide in 2012, accounting for about 13% of total cancer diagnoses [1]. It was estimated to have caused 1.6 million deaths, and resulted in 34.7 million disability-adjusted life-years in 2013. In addition, it was the most common cause of cancer death globally, in both developing and developed countries [2].

Although early-stage lung cancer can be treated by surgery, the reduction in the respiratory function after lung resection significantly worsens the physical health and quality of life of the patients [3, 4]. Recently, many patients, including those with inoperable tumors, undergo radiotherapy such as stereotactic body radiotherapy (SBRT) [5–7], which has been shown to be as effective as resection for stage I lung cancer [8, 9].

In this aging society, the proportion of elderly patients being diagnosed with lung cancer is increasing. Moreover, their decreased physical ability, and the presence of comorbidities such as congestive heart failure, cerebrovascular disease, chronic pulmonary disease, and chronic renal disease impedes effective treatment. An increase in the number of comorbidities is directly correlated with increased mortality rates in patients [10].

An increasing number of lung cancer patients are being treated using proton beam therapy (PBT) with or without chemotherapy [11–15]. The advantage of PBT over conventional radiotherapy or SBRT using X-ray irradiation is that the former can deliver a more concentrated dose of radiation to tumors and a lower dose to normal tissue [16–18]. However, few reports have been published regarding the use of PBT in the treatment of lung cancer patients aged ≥80. Thus, the purpose of the present study was to evaluate retrospectively the efficacy and safety of PBT for elderly patients with lung cancer.

## Methods

### Ethics statement

This retrospective study was approved by the ethics committees of our institution (approval number: D17–31). The study was conducted in accordance with the Declaration of Helsinki.

### Patients

The present study included patients who were diagnosed with lung cancer and treated with PBT between January 2009 and 2015 at the Southern Tohoku Proton Therapy Center. Patients were retrospectively recruited from our database. The clinical stage of the patients’ lung cancer (Union for International Cancer Control, 8th edition) was determined using computed tomography (CT) and positron emission tomography (PET)-CT. Written informed consent was obtained from all of the patients. The inclusion criteria were as follows: patients aged ≥80 years when the patint received PBT; a solitary lung tumor; pathologically proven non-small cell lung cancer (NSCLC); a World Health Organization performance status of 0–2; no lymph node metastasis; and the absence of distant other organ metastasis or other sites of uncontrolled cancer. Any patients who received concurrent chemotherapy, and those with interstitial pneumonitis were excluded. The judgment as to the operability was made by a conference of lung surgeons. All operable patients who received PBT refused to receive lung surgery.

### Proton beam therapy

Treatment planning for PBT was based on three-dimensional CT images that were taken at 2 mm intervals in the exhalation phase while using a respiratory gating system (Anzai Medical, Tokyo, Japan). A custom-indexed vacuum-lock bag was used to immobilize the patients. An XiO-M (CMS Japan, Tokyo, Japan; and Mitsubishi Electric) treatment planning system was used to calculate the dose distributions for PBT. The gross tumor volume (GTV) included the lung tumor. The clinical target volume (CTV) was defined as the GTV plus a margin of 0.5 cm. The planning target volume (PTV) was the CTV plus a 0.5 cm margin. The proton energy levels of 150 MeV and 210 MeV for 2–3 portals, and a spread-out Bragg peak were tuned as much as possible until the PTV was exposed to a 90% isodose of the prescribed dose. Proximal margins, distal margins, and smearing margins were calculated using strategy 2, which Moyers et al. reported [19]. The PBT system at our institute (Proton Beam System, Mitsubishi, Tokyo, Japan) used a synchrotron and a passive scattering method in which a proton beam passes a bar ridge filter, a range shifter, and a customized compensator before entering the patient. The treatment was administered during the exhalation phase using a respiratory gating system. A multileaf collimator, which consisted of 40 iron plates with a width of 3.75 mm, and which could be formed into an irregular shape, was used. Daily front and lateral X-ray imaging was used for positioning. The PBT schedule was 66 Gy relative biological dose effectiveness (RBE) in 10 fractions over 2 weeks for peripheral lung tumors, and 80 Gy (RBE) in 25 fractions over 5 weeks for centrally located lung tumors. Patients with lung tumors that were located near the large intestine or small intestine received 60.0–79.2 Gy (RBE) in 25–33 fractions over 7 weeks.

### Evaluation and follow-up

Comorbidities were evaluated as previously reported by Charlson et al. [10], and shown in the index. All patients underwent either CT or PET-CT to evaluate the initial tumor response within three months after the completion of treatment. The follow-up interval was every 1–3 months for the first year and every 3–6 months thereafter. Pneumonitis, excluding infection, was evaluated using the Common Terminology Criteria for Adverse Events version 4.0 [20].

### Statistical analyses

All statistical analyses were performed using the IBM SPSS Statistics software program (version 22, SPSS Inc., Chicago, IL, USA). The overall survival (OS) time was defined as the time between the start of PBT and the time of the last follow-up examination or death. The Kaplan–Meier method and a log rank test were used to estimate the survival probability and compare survival, respectively. The relationships between the occurrence of lung toxicities and the dose volume histogram factors were examined using the Mann–Whitney *U* test. The dosimetric factor of lung was examined in 2Gy per fraction using lung alpha/beta ratio was 3.1 [21]. Lung V20 (ratio of lung volume irradiated at leaset 20 Gy) and mean lung dose using a dose volume histogram. The ratio of the lung volume spared from 0.05 Gy (RBE) dose (total lung volume minus lung volume irradiated at least 0.05 Gy [RBE]) was also examined, as Tsujino et al. [22] reported that the lung volume speared from low irradiation dose. All *p*-values were two sided, and *p* values of < 0.05 were considered to indicate statistical significance.

## Results

### Patients

The initial study population included 78 patients aged ≥80 years who had received PBT for a lung tumor. Patients were excluded from the analysis for the following reasons: lymph node metastasis (*n* = 28); distant other organ metastasis (*n* = 3); interstitial pneumonitis (*n* = 4); and absence of NSCLC pathology (*n* = 8). Thus, the characteristics of 35 patients, including 25 (71%) with clinically inoperable NSCLC, were analyzed (Table 1). All patients completed treatments. The cohort comprised 26 men and 9 women, with a median age of 82 years (range: 80–87 years). The comorbidity index of 30 patients (86%) was ≥1. The median follow-up time was 34 months (range: 10–72 months). The median dose of PBT was 80.0 Gy (range: 60.0–80.0 Gy [RBE]).

**Table 1:** The patient characteristics (*n* = 35)

Characteristics	
Age (years)	
Median (range)	82 (80–87)
Gender	
Male	9 (26%)
Female	26 (74%)
Performance status	
0	14 (40%)
1	15 (43%)
2	6 (17%)
Comorbidity index	
0	5 (14%)
1	17 (48.5%)
2	8 (23%)
3	3 (8.5%)
4	2 (6%)
Operable or inoperable	
Operable	10 (29%)
Inoperable	25 (71%)
Follow-up time (months)	
Median (range)	34 (10–72)
T category ^a^	
T1	13 (37%)
T2	13 (37%)
T3	8 (23%)
T4	1 (3%)
Stage ^a^	
I	21 (60%)
II	13 (37%)
III	1 (3%)
Tumor location	
Right upper lobe	4 (11%)
Right middle lobe	2 (6%)
Right lower lobe	8 (23%)
Left upper lobe	14 (40%)
Left lower lobe	7 (20%)
Histopathology	
Squamous cell carcinoma	17 (48.5%)
Adenocarcinoma	17 (48.5%)
Non-small cell lung cacner	1 (3%)
Diameter of lung tumor (mm)	
Median (range)	32.0 (10.0–67.0)
Total dose (Gy (RBE))	
Median (range)	80.0 (60.0–80.0)

*Abbreviations: RBE* relative biological dose effectiveness

^a^TNM classification of malignant tumor 8th edition (Union for international cancer contorol)

### Survival rate

**Fig. 1:**
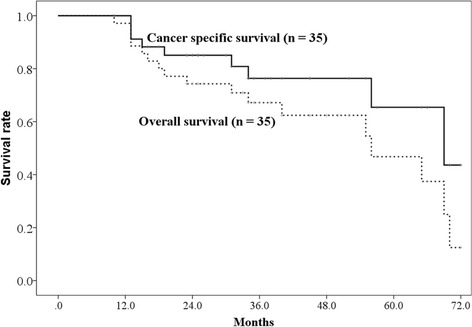
The overall survival rate and disease-specific survival rate. The 3-year overall survival rates and cancer-specific survival rates were 66.7% (95% CI: 50.3–83.1%) and 76.3% (95% CI: 60.4–92.2%) respectively

**Fig. 2:**
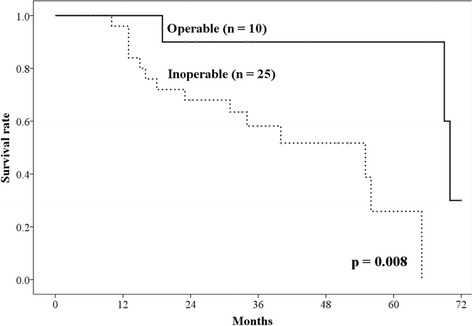
The overall survival rate of patients with operable and inoperable tumors. The 3-year overall survival rates of patients with operable and inoperable tumors were 90%, and 57.1%, respectively (*p* = 0.021)

All patients were followed for at least 23 months or until their death. The 1-year, 2-year, and 3-year OS rates were 97.1% (95% CI: 91.6–100%), 74.3% (95% CI: 59.8–88.8%), and 67.2% (95% CI: 50.3–83.3%), respectively (Fig. 1). The median survival time was 56 months (95% CI: 33.1–78.9 months). The 3-year cancer specific survival rate was 76.3% (95% CI: 60.4–92.2%). Nine patients died due to lung cancer (metastasis, *n* = 7; local recurrence, *n* = 2), and 8 patients died due to other causes (another newly diagnosed cancer after PBT, *n* = 4; other diseases, *n* = 4). The 3-year OS rate was significantly different between operable and inoperable patients (90% vs 58.2%, *p* = 0.008) (Fig. 2).

### Failure patterns

**Fig. 3:**
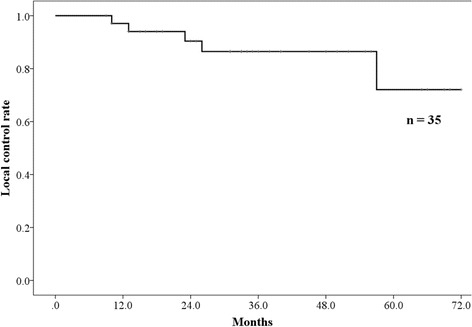
The local control rates. The 3-year local control rate was 85.7% (95% CI: 72.0–99.4%)

The 3-year local control rate was 86.5% (95% CI: 74.0–99.0%) (Fig. 3). Four patients had local recurrence at 9–33 months. Two had lymph node metastasis, 6 had lung metastasis outside of the PBT field. Regarding other organ metastasis, 1 patient had liver metastasis, 1 patient had brain metastasis, and 4 had pleural dissemination.

### Toxicities

There were no cases involving grade 4 or 5 toxicities after PBT treatment (Table 2). The toxicities of the 35 patients included 2 (5.6%) cases of grade 2 pneumonitis, 4 (11.1%) with grade 2 rib fractures, and 1 (2.8%) with grade 3 dermatitis radiation. A high ratio of V 20 (18.7% vs 10.9%, *p* = 0.030), high dose of mean lung dose (12.4Gy vs 7.5Gy *p* = 0.030), and low ratio of lung volume spared from 0.05 Gy (RBE) dose (62.9% vs 77.7%, *p* = 0.030) were significantly correlated with the occurrence of grade2 pneumonia.

**Table 2:** Toxicities

Toxicities	Grade 0	Grade 1	Grade 2	Grade 3	Grade 4/5
Pneumonitis	2 (6%)	31 (88%)	2 (6%)	0	0
Rib fracture	26 (74%)	5 (14%)	4 (12%)	0	0
Dermatitis radiation	5 (14%)	22 (63%)	7 (20%)	1 (3%)	0

## Discussion

To the best of our knowledge, this is the first report of PBT for elderly patients (≥80 years of age) with lung cancer. In an aging society, treatment which can reduce toxicities is particularly important because elderly patients are less able to tolerate toxicities when compared to their younger counterparts.

The SBRT was one of the treatment choices for elderly patients with lung cancer, with a 3-year OS rate was 59–83% [5–7]. The 3-year OS rate in the present study was slightly worse than in previous reports, possibly because our patient cohort was older and had larger sized tumors than previous reports. However, grade 3 or worse pneumonitis was not observed in the present study, in contrast to previous reports of a 7.7% incidence of grade 3 and 4 pneumonitis following SBRT treatment [7], and a 2.4% grade 3 pneumonitis in another study by Li et al. [5]. This was attributed to the ability of PBT to irradiate the malignant tissue with greater specificity than X-ray irradiation while sparing normal tissue. In fact, Barriger et al. previously reported that the mean lung dose and lung V20 were correlated with radiation pneumonitis after SBRT [23]. In the present study, we also obtained similar results when the biological effective dose adjusted. Moreover, the low ratio of lung volume spared from 0.05 Gy (RBE) dose was correlated with occurrence of pneumonia in the present study. Regarding the dose volume with SBRT versus that with PBT for early lung cancer, Kadoya et al. reported that the PBT dosage was able to be significantly reduced compared with SBRT [16]. This suggests tha PBT can deliver the same dose while sparing more normal lung volume from irradiation than SBRT, which may lead to a lower occurrence rate of pneumonia and safer treatment relative to SBRT, even in elderly patients.

For elderly patients, lung resection, including lobectomy, is also a treatment choice for lung cancer. A comparison of SBRT and lung resection showed that the OS of patients receiving surgery was better than that of those receiving SBRT in an unadjusted population [24–26]. One reason for this may be that patients receiving SBRT tended to be significantly older and had lower OS. In fact, Paul et al. reported that the OS in the SBRT group with ≤2 cm lung tumors was the same as that of the surgery group in a propensity score matched analysis [26]. In contrast, Shirvani et al. on comparing five treatment strategies in elderly NSCLC patients using a propensity score matched analysis reported that the survival of all patients who received SBRT was similar to that of patients who underwent lobectomy [25]. Their report also found that early mortality of elderly patients was the lowest in the SBRT group. As mentioned previously, PBT treatment irradiates less of the normal lung than SBRT. This suggests that PBT for elderly patients may achieve even lower mortality rates than SBRT and surgery, although no studies to date of have directly compared the effect of PBT with other treatment options.

The 2-year OS of PBT for early stage lung cancer was previously reported to be 31–80% [11, 13, 14]. The OS rate in the present study was not marked worse than in those previous reports, even though our patient cohort only comprised of elderly patients. This suggests that PBT for elderly patients (≥80 years of age), including those with inoperable cancer, is as feasible and effective as in younger lung cancer patients.

Degradation of the Bragg peak occurs when PBT is performed for lung cancer [27]. To check the areas irradiated by the proton beam, PET-CT was conducted after PBT. Changing the treatment plan was not needed in any patients based on PET-CT findings in our institution.

This study had several limitations, including the small sample size and the retrospective design. However, as only a few reports have described PBT treatment in elderly lung cancer patients, we believe that the results of the present study are essential and warrant further research.

## Conclusions

These findings suggest that PBT for elderly patients with NSCLC is feasible, and can be considered as one of the treatment choices for elderly lung cancer patients.

## Abbreviations

CT: Computed tomography
CTV: Clinical target volume
GTV: gross tumor volume
NSCLC: Non-small cell lung cancer
OS: Overall survival
PBT: Proton beam therapy
PET: Positron emission tomography
PTV: Planning target volume
RBE: Relative biological effectiveness
SBRT: Stereotactic body radiotherapy
